# Characterization of Polyphenolic Compounds and Antioxidant Activities of *Tagetes* Flowers With Varying Treatments Using LC‐ESI‐QToF‐MS/MS

**DOI:** 10.1002/fsn3.71047

**Published:** 2025-10-03

**Authors:** Ayesha Siddiqa, Adnan Khaliq, Muhammad Tauseef Sultan, Muhammad Farhan J. Chugthai, Samreen Ahsan, Waseem Khalid, Hafiz Suleria

**Affiliations:** ^1^ Institute of Food Science and Technology Khawaja Fareed University of Engineering and Information Technology Rahim Yar Khan Punjab Pakistan; ^2^ School of Agriculture, Food and Ecosystem Sciences University of Melbourne Australia; ^3^ Department of Human Nutrition, Faculty of Food Science and Nutrition Bahauddin Zakariya University Multan Pakistan; ^4^ Department of Organic Chemistry, Faculty of Chemical Sciences and Technologies University of Castilla La Mancha Ciudad Real Spain

**Keywords:** bioactive compounds, HPLC‐DAD, LC‐ESI‐QTOF‐MS/MS, marigold, *Tagetes*, ultrasonication

## Abstract

*Tagetes erecta* is a valuable medicinal plant, with its flowers being edible and recognized for numerous health benefits attributed to its bioactive polyphenolic compounds. Despite the increasing interest in polyphenols from medicinal plants, limited studies have explored the impact of processing and extraction methods on the bioactive compounds of *Tagetes* flowers, especially those often discarded after a single use. This study investigated and compared the efficacy of extraction methods on polyphenolic compounds by varying methods of drying and extraction. The total phenolics and their antioxidant activities were measured using free radical scavenging, reducing power, and ferrous ion‐chelating assays. LC‐ESI‐QTOF‐MS/MS and high‐performance liquid chromatography (HPLC) coupled with a diode array detector (DAD) were used for the identification and quantification of individual phenolic compounds. The higher total phenolic content and flavonoid content were observed in freeze‐dried samples of marigold upon ultrasound extraction using ethanol (74.57 mg GAE/g). Antioxidant potential in various assays: DPPH (18.49 mg AAE/g), ABTS (32.14 mg AAE/g), RPA (66.39 mg TE/g), TAC (70.25 mg AAE/g) were also observed highest (70% ethanol), while FRAP (59.76 mg AAE/g), FICA (2.17 mg EE/g), and OH^−^ (267 mg TE/g) in (70% acetone) ultrasonicated extracts of freeze‐dried samples. FT‐IR of various freeze‐dried (FD) extract samples were compared with the raw powder of *Tagetes*, which exhibited similar functional groups, confirming that ultrasonication had no adverse effect on the functional bioactive compounds. Liquid chromatography with mass spectrometry (LCMS/MS) analysis tentatively identified 33 polyphenolic compounds, including phenolic acids (13), carotenoids (3), flavonoids (14), and other polyphenols in conventional and ultrasound extract samples. High‐performance liquid chromatography (HPLC‐DAD) quantification of freeze‐dried samples showed phenolic acids to be higher for samples [MFEU (85.28 mg/g) > MFAU (70.71 mg/g)] extracted through ultrasound‐assisted extraction. These findings indicate that the application of ultrasound‐assisted extraction (UAE), in combination with suitable drying methods and solvent systems, can substantially enhance the recovery of functional compounds from *Tagetes flowers*, its potential utilization in nutraceuticals and functional food products, while also contributing to the reduction of plant‐based waste.

## Graphical abstract

This study demonstrates the effect of drying and extraction methods with varying solvents on polyphenolic compounds, as well as the potential antioxidant and radical scavenging activities of *Tagetes* full bloom flowers. Results showed the higher phenolic content and antioxidant activities in freeze‐dried (FD) samples extracted with ethanol in the ultrasound‐assisted (UAE) method. Polyphenolic compounds characterization and quantification also support the premise that UAE is an efficient approach for bioactive compounds recovery, supporting the valorization of *Tagetes* flowers in nutraceutical applications.

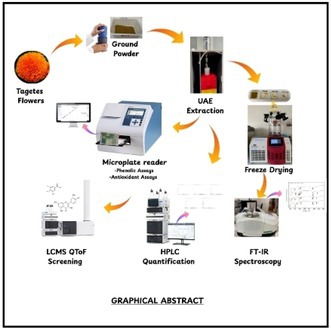

## 1 Introduction

With increasing consumer awareness and interest in the antioxidant potential and health benefits of bioactive compounds, medicinal plants have garnered considerable attention from the pharmaceutical and nutraceutical sectors. Numerous phytochemicals present in *Tagetes* plants are widely explored for utilization in traditional medicines (Estrada et al. 2024). Wild edible flowers (including *Tagetes*) flagged significant bioactive reservoirs consisting of phenolic compounds, carotenoids, flavonoids, and other polyphenols with potential antioxidant activities (Hegde et al. 2023). Among various phytochemicals, phenolic compounds are noteworthy due to their myriad health benefits against various chronic and age‐related disorders.

*Tagetes erecta* (Marigold) flowers have been identified as safe and rich in therapeutic substances. It is a prominent economic and industrial plant that is predominantly cultivated for commercial use (Chitrakar et al. 2019). An estimated six million tonnes of marigold flowers are produced globally (Singh et al. 2020) for commercial applications in food, feed, pharma, cosmetics, and textile (dyeing) (Wu et al. 2023). The global market demand for *Tagetes* lutein continues to increase, potentially approaching USD 357 million by 2022 (Ma 2017), and is expected to reach USD 405 million by 2027 (Saha et al. 2020). Generally cultivated flowers are procured for many religious and cultural events and subsequently discarded post‐event and become a source of pollution. Its environmental repercussions are becoming one of the serious global issues due to inappropriate solid waste disposal, poor management systems, and waste utilization strategies. This is a serious sustainable development issue highlighted by SDG 12 due to its impact on the environment and human health. Organic waste such as festive flowers (*Tagetes*) (Mir et al. 2019), leaves, and shells (coconut) contribute to solid waste (Adhikary and Vishwavidyalaya 2020). It has been estimated that over 5 billion tonnes of floral waste, primarily sourced from temples, have been collected from various regions of the country, with an increase during festival periods. This subsequently contributes to multiple forms of pollution. The blooms are utilized once and subsequently discarded. India currently generates 2,785,000 metric tonnes of flowers. The floriculture trade is anticipated to expand at a compound annual growth rate (CAGR) of 20.1% from 2019 to 2024 (Goel and Kulshrestha 2024). The growing need is a significant problem; thus, it is imperative to explore local sources and improve extraction methods to achieve improved yields, lower prices, and augment shelf stability for commercial applications.

*Tagetes erecta* L. is a commercially cultivated plant native to Pakistan (Baig et al. 2021). In this study, locally grown *Tagetes erecta* L. flowers were investigated fully for their bioactive phenolic compounds by varying drying methods (freeze drying, oven drying), extraction methods (Ultrasound‐assisted extraction, conventional solvent extraction), and solvents (ethanol, acetone, and hexane). The slow heat transfer mechanism in the oven drying process may cause denaturation of polyphenolic compounds (polyphenol oxidase) involved in the degradation of antioxidant compounds (Rabeta and Vithyia 2013), while the freeze‐drying method protects phenolic compounds and they may get more concentrated on ice trap (Subbiah, Duan, et al. 2023). The phenolic content of the *Tagetes* flower was comprehensively investigated by using set methods of our group employing various phenolic assays including TPC, TFC, and TCT methods based on different mechanisms (Suleria et al. 2020). The Folin–Ciocalteu method is a non‐specific analytical method and may overestimate the phenolic content due to the presence of other powerful reducing agents. The FCR method may also detect certain peptides and result in higher TPC values (Mosele et al. 2016; Ma et al. 2021). Therefore, 11 different assays were performed to fully investigate samples, drying, and extraction methods' impact on phytochemicals. The antioxidant potential of *Tagetes* flower sample extracts was investigated by employing various antioxidant assay methods based on different mechanisms. In this study, seven different antioxidant assays including DPPH, FRAP, ABTS, RPA, ^•^OH‐RSA, FICA, and TAC were performed to evaluate the radical scavenging activities (RSA), metal chelating, hydrogen atom donating, and reducing potential of flower extracts. Antioxidant compounds protect the human body owing to their potential to reduce oxidative stress by scavenging free radicals, binding the metals, and supplying hydrogen atoms to reactive systems (Kıran et al. 2023). *Tagetes* flowers are rich sources of bioactive compounds including phenolics, flavonoids, and carotenoid compounds, particularly lutein imparting antioxidant activities, making it a complex system. Thus, using a combination of antioxidant and radical scavenging assays provides a more comprehensive understanding of antioxidant activities. The rationale of the study was based on limited research on the quantification of polyphenolic compounds of *Tagetes* for nutraceutical applications. To the best of our knowledge, while numerous studies have investigated the impact of drying and extraction methods on the phenolic compounds of various medicinal plants, few have systematically explored these effects on the polyphenolic profile of flower biomass typically discarded after a single use. The current study focuses on the valorization of bioactive polyphenolic compounds and their antioxidant potential under varying treatments, with the aim of optimizing extraction methods for nutraceutical applications and promoting plant waste reduction. Further, quantification was performed for a more specific characterization of phenolic compounds by using an advanced highly sensitive liquid chromatography technique coupled with electrospray‐ionization and quadrupole time‐of‐flight mass spectrometry [LC‐ESI‐QToF‐MS/MS] and their quantification by HPLC‐DAD. This thorough investigation offers essential insights into the bioactive potential of these underutilized non‐food, waste valuable products such as edible flowers, establishing a foundation for their prospective transformation into functional foods, nutraceuticals, or pharmaceutical products.

## 2 Materials and Methods

### 2.1 Sample Collection and Preparation

*Tagetes* flowers were collected at the full blooming stage from the local nurseries of KFUEIT, Rahim Yar Khan, Pakistan. The *Tagetes* flower species was verified by a horticulture expert from the Department of Horticulture, Agriculture Engineering and Sciences, Khwaja Fareed University of Engineering and Information Technology. The samples were dried in an air‐based food dehydrator (Model No. DI‐2400) under specific conditions of temperature 40°C, time 3–4 h (Badmus et al. 2019), and freeze drier (Thermo scientific freezer) at −60°C for 72 h at The University of Melbourne, Australia. Dried flower petals were ground to a fine powder using a grinder (Cuisinart Nut and Spice grinder), labeled, and kept separately in air‐tight polyethylene bags and stored at 4°C.

### 2.2 Chemicals

Chemicals used for extraction include organic solvents: ethanol, acetone, hexane, and diethyl ether (C_2_H_5_)_2_O used for saponification to remove impurities from oleoresin. Other chemicals used for purification include potassium hydroxide (KOH) and sodium sulfate anhydrous (Na_2_SO_4_) purchased from Sigma Aldrich (Castle Hill, NSW, Australia). Phenolic and antioxidant assays were performed by using chemicals including sodium carbonate anhydrous (Na_2_CO_3_), Folin & Ciocalteu's phenol reagent, vanillin, methanol, catechin hydrate, 2,2′‐diphenyl‐1‐picrylhydrazyl (DPPH), aluminum chloride (AlCl_3_) hexahydrate, sodium acetate (CH_3_COONa), vanillin, iron (III) chloride (FeCl_3_), 2,4,6‐tripyridyl‐s‐triazine (TPTZ), sodium hydroxide pellet (NaOH), potassium persulfate (K_2_S_2_O_8_), iron (II) sulfate heptahydrate (FeSO_4▪_7H_2_O), 3‐hydroxybenzonic acid, sodium phosphate dibasic heptahydrate (Na_2_HPO_4▪_7H_2_O), sodium phosphate monobasic heptahydrate (Na_2_HPO_4▪_7H_2_O), Potassium ferricyanide (III) (K_3_[Fe(CN)_6_]), trichloroacetic acid (TCA), 3‐(2‐pyridyl)‐5,6‐diphenyl‐1,2,4‐triazine‐p–p′‐‐disulfonic acid monosodium salt hydrate (Ferrozine), sodium hydroxide solid (NaOH), trisodium phosphate (Na_3_PO_4_), and ammonium molybdate [(NH_4_)_6_ Mo_7_O_24_]. All chemical solvents used were ethanol, methanol, sulfuric acid (H_2_SO_4_), acetic acid glacial, hydrogen peroxide (H_2_O_2_), hydrochloric acid (HCl), and the standards used: gallic acid monohydrate, L‐ascorbic acid, quercetin, catechin, trolox, ethylene diamine tetra acetic acid (EDTA) were also purchased from Sigma Aldrich (Castle Hill, NSW, Australia) and 98% sulfuric acid from RCI Lab scan (Rongmuang, Thailand).

### 2.3 Extraction Methodologies

#### 2.3.1 Conventional Solvent Extraction (CSE)

For conventional solvent extraction, samples were prepared by following the method of Subbiah, Ebrahimi, et al. (2023) by taking 5 g of sample into 50 mL of 70% selected solvents (ethanol, hexane, and acetone). Samples were mixed well and incubated in a Ratek orbital shaker (OM11, Ratek Orbital Shaking Incubator, VIC, Australia) at 120 rpm for 12 h at 28°C in the dark. After incubation, samples were centrifuged at 8000 rpm for 15 min at 4°C by using a refrigerated centrifuge (Hettich, ROTI NA380R, Tuttlingen, Baden‐Württemberg, Germany), and the supernatant was pooled in labeled test tubes and stored at −18°C for further analysis.

#### 2.3.2 Ultrasound‐Assisted Extraction (UAE)

Extraction with pre‐treatment as ultra‐sonification was performed by employing the method reported by (Shi et al. 2023). Ultrasound‐assisted extraction was performed by employing the optimal conditions: Amplitude 40%; Time: 5 min by using Ultrasonifier (Branson, Digital Sonifier model 450). All the samples were carefully labeled and mixed with different solvents at a ratio of 1:10 (w/v) and incubated in Ratek orbital shaker (OM11, Ratek Orbital Shaking Incubator, VIC, Australia) at 120 rpm for 12 h at 28°C in the dark. After overnight incubation, samples were centrifuged at 8000 rpm for 15 min at 4°C by using a refrigerated centrifuge (Hettich, Roti NA380R, Tuttlingen, Baden‐Württemberg, Germany), and the supernatant was pooled in labeled test tubes and stored at −18°C for further analysis.

Prepared extracts of various treatments are classified (Table 1) for further analysis.

**TABLE 1:** Sample treatment plan with treatment conditions and abbreviations of samples based on extraction method, solvent type, and drying techniques.

Extraction methods	Solvents	Oven dried samples	Freeze dried samples
Conventional solvent extraction (CSE)	Ethanol	MOEC	MFEC
	Acetone	MOAC	MFAC
	Hexane	MOHC	MFHC
Ultrasound assisted extraction (UAE)	Ethanol	MOEU	MFEU
	Acetone	MOAU	MFAU
	Hexane	MOHU	MFHU

*Note:* Extraction Treatments are abbreviated as follows: CSE: Conventional solvent extraction; UAE: Ultrasound‐assisted extraction; Marigold samples with different treatments, abbreviations are: MFAC, marigold freeze dried‐acetone based conventional extracts; MFAU, marigold freeze dried‐acetone based ultrasonified extracts; MFEC, marigold freeze dried‐ethanol based conventional extracts; MFEU, marigold freeze dried‐ethanol based ultrasonified extracts; MFHC, marigold freeze dried‐hexane based conventional extracts; MFHU, marigold freeze dried‐hexane based ultrasonified extracts; MOAC, marigold oven dried‐acetone‐based conventional extracts; MOAU, marigold oven dried‐acetone‐based ultrasonified extracts; MOEC, marigold oven dried‐ethanol‐based conventional extracts; MOEU, marigold oven dried‐ethanol‐based ultrasonified extracts; MOHC, marigold oven dried‐hexane‐based conventional extracts; MOHU, marigold oven dried‐hexane‐based ultrasonified extracts.

### 2.4 Estimation of Phenolic Content and Antioxidant Assays

The polyphenolic content and antioxidant assays were performed to evaluate the phenolic content and antioxidant activities of *Tagetes* flower samples of selected stages. For polyphenolic content determination of TPC, TFC and TCT and antioxidant activities seven assays including DPPH, FRAP, ABTS, TAC, RPA, FICA, and ^•^OH^−^ were performed by using Multiskan Go microplate photometer (Thermo Fisher Scientific, Waltham, MA, USA) for absorbance measurement and data calculation, further statistical analysis was performed by using Minitab.

#### 2.4.1 Total Phenolic Content (TPC)

The total phenolic content of *Tagetes* was measured using the reducing Folin Ciocalteu Reagent following the method of (Manivannan et al. 2021). For this purpose, the sample extract (25 μL) was mixed with 25 μL of FC reagent + 200 μL of water and allowed to stand for reaction (5 min at 25°C). Sodium carbonate solution was added to the above reaction mixture, resulting in a blue color as the endpoint. Reaction samples were allowed to stand at 25°C for 60 min in the dark and then subjected to a UV–Vis spectrophotometer (Thermo Fisher Scientific, Waltham, MA, USA) for absorbance measurement at a wavelength of 765 nm against gallic acid standard concentration ranging from 0 to 200 μg/mL. TPC was calculated and expressed as mg GAE/g of dry weight.

#### 2.4.2 Total Flavonoid Content (TFC)

The total flavonoid content in marigold sample extracts was determined by employing the method adopted by (Siriamornpun et al. 2012), marigold extract sample (80 μL) was mixed with an equal amount (80 μL) of sodium nitrite solution and 120 μL of 2% aluminum chloride. The sample extract was then mixed with 120 μL of sodium acetate and incubated for 2.5 h. The reaction mixture was vortexed for proper mixing, and the absorbance was measured at 440 nm with standard Quercetin. The results for TFC are expressed in mg QE/g of dry weight.

#### 2.4.3 Total Condensed Tannin (TCT)

Total condensed tannins were measured by the vanillin assay method reported by Suleria et al. (2020). Marigold flower (MFP) extracts were diluted with extracting solvents (1:10) and measured 25 μL in wells of the microplate. Further, 150 μL of vanillin solution (4%) was mixed with 25 μL of sulfuric acid (32% v/v, H_2_SO_4_) in a 96‐well plate. For the quantification of TCT, the reaction mixture was incubated in the dark for 15 min at 25°C, and absorbance was measured at 500 nm. The standard curve based on catechin (0–1000 μg/mL) was constructed, and TCT was expressed as the mass of catechin equivalent per gram (mg CE/g) dry weight.

#### 2.4.4 2,2′‐Diphenyl‐1‐Picrylhydrazyl (DPPH) Assay

Antioxidant assays of different marigold extracts were performed by measuring the DPPH radical scavenging activity, employed by (Li et al. 2007), with slight modification in the method. Prepared extract samples at various concentrations from 50 to 100 μL aliquots were measured in labeled test tubes. The volume of aliquots was adjusted to 1000 μL by using a methanol solution for the standard curve. 25 μL of sample extract was added into 275 μL of 0.1 mM DPPH solution and was prepared and mixed well by shaking before incubation (dark cool place temp. 30°C) for 30 min. As radical DPPH has the potential to capture hydrogen from the sample extract, it is indicated by its color transformation from purple to yellow. Samples were then subjected to a UV–Vis spectrophotometer at a wavelength of 517 nm against the ascorbic acid standard curve and results were expressed as the mg AAE/g dry weight of *Tagetes* powder sample.

#### 2.4.5 Ferric Ion Reducing Antioxidant Potential (FRAP) Assay

The ferric ion‐reducing antioxidant potential (FRAP) of *Tagetes erecta* was measured by the antioxidant activities measuring method employed by Ali et al. (2021). The FRAP reagent reaction mixture (dye) was prepared using 2,4,6 Tris (2‐pyridyl)‐s‐triazine (10 mM), Ferric chloride (20 mM), and sodium acetate (300 mM) in 1:1:10. After reagent mix preparation, 20 μL of marigold extract sample was mixed with 280 μL of FRAP dye solution and incubated (OM11, Ratek Orbital Shaking Incubator) in the dark for 10 min at 37°C. After incubation, absorbance was recorded at 593 nm using a UV‐spectrophotometer. The standard curve for ferric ion‐reducing antioxidant potential was derived by varying the concentration of ascorbic acid (0–200 μg/mL), and the results were expressed as mmol of AAE/g DW of *Tagetes* powder sample.

#### 2.4.6 2,2′‐Azino‐Bis‐3‐Ethylbenzothiazoline‐6‐Sulfonic Acid (ABTS ^+^) Assay

The free radical scavenging activity of MFP extract samples was measured by using 2,2′‐azino‐bis‐3‐ethylbenzothiazoline‐6‐sulfonic acid (ABTS^+^) decolorization assay method (Shi et al. 2024). The ABTS^+^ dye stock solution was prepared by using 2,2′‐azino‐bis‐3‐ethylbenzothiazoline‐6‐sulfonic acid solution (7 mM) and potassium persulfate (K_2_S_2_O_8_) (140 mM). Further, ABTS^+^ free radical solution was prepared from stock dye solutions by mixing 3 mL of ABTS with 52.8 μL of potassium persulfate solution and incubated for 16 h in the dark. After incubation, ABTS^+^ dye (A_734_ = 0.61) was diluted with ethanol (45 mL) before preparing the reaction mixture in a well plate. The reaction mixture was prepared by taking 10 μL of extract and 290 μL of ABTS^+^ dye in a 96‐well plate, incubated for 6 min at 25°C. Radical scavenging activity was determined by measuring absorbance at 734 nm in a microplate reader (Thermo Fisher Scientific, Waltham, MA, USA). The standard curve based on ascorbic acid (0–150 μg/mL) was constructed, and results were expressed as the mass of ascorbic acid equivalent (AAE) per gram (mg AAE/g) dry weight of *Tagetes* powder sample.

#### 2.4.7 Ferrous Ion Chelating Activity (FICA) Assay

Ferrous ion chelating activity (FICA) assay is based on the chelating principle in which free metal ions form bonds with coordinate bonds to form stable complexes. FICA assay was performed by following the method of Shi et al. (2024) to assess the chelating potential of marigold flower sample extracts. Chemical reagents were prepared by using ferrous chloride (2 mM) and ferrozine (5 mM); further solutions were diluted to 1:15 and 1:6, respectively, with Milli‐Q water. For the reaction mixture, 15 μL of each extracted sample was measured with 85 μL of water, further mixed with 50 μL of diluted ferrous chloride and ferrozine solutions into microplate wells, and incubated for 10 min at 25°C. Ferrous ion chelating activity was determined by measuring absorbance at 562 nm in a microplate reader. The standard curve based on ethylenediaminetetraacetic acid (EDTA) (0–50 μg/mL) was constructed, and results were expressed as mass of EDTA equivalent per gram (mg EE/g) dry weight of *Tagetes* powder sample.

#### 2.4.8 Hydroxyl Radical Scavenging Assay (^•^ OH‐RSA)

The hydroxyl radical scavenging activity (^•^OH‐RSA) assay based on the principle of the Fenton reaction (Salgado et al. 2013) was performed to assess the potential of marigold flower extract samples to scavenge reactive species hydroxyl radicals (^•^OH). In this assay method, 50 μL of marigold extract samples were mixed with 50 μL of 6 mM iron (II) ferrous sulfate heptahydrate (Fe_2_SO_4_.7H_2_O) and 50 μL of 6 mM hydrogen peroxide (H_2_O_2_) into microplate wells and incubated for 10 min at 25°C. After incubation, 50 μL of 6 mM hydroxybenzoic acid (C_7_H_6_O_3_) was added to the reaction mixture and again incubated for 10 min at 25°C. The hydroxyl radical scavenging activity was determined by measuring absorbance at 510 nm in a microplate reader. The standard curve based on trolox (0–1000 μg/mL) was constructed and results were expressed as the mass of trolox equivalent per gram (mg TE/g) dry weight of *Tagetes* powder sample.

#### 2.4.9 Reducing Power Assay (RPA) Assay

Reducing power assay (RPA) based on the reduction principle donates its hydrogen to convert ferricyanide (Fe ^3+^) to ferrocyanide (Fe ^2+^). RPA was performed by measuring 10 μL of marigold flower extract samples into wells of a microplate to assess their reducing power. Buffer A [0.2 M Sodium phosphate dibasic heptahydrate (Na_2_HPO_4_▪7H_2_O)] and Buffer B [0.2 M Sodium phosphate monobasic monohydrate (Na_2_HPO_4_▪H_2_O)] were mixed to prepare the solution mixture Buffer C (pH 6.6). 25 μL of buffer C solution and 25 μL of 1% potassium ferricyanide were mixed into sample extracts in wells and incubated in the dark for 20 min at 25°C. After incubation, 25 μL of the 10% trichloroacetic acid (TCA), 85 μL of Milli‐Q water, and 8.5 μL of 0.1% ferric chloride (FeCl_3_) were further added to the reaction mixture, and the microplate was again incubated for 10 min at 25°C. Reducing power was determined by measuring absorbance at 750 nm in a microplate reader. The standard curve based on ascorbic acid (0–500 μg/mL) was constructed, and results were expressed as the mass of ascorbic acid equivalent per gram (mg AEE/g) dry weight of *Tagetes* powder sample.

#### 2.4.10 Total Antioxidant Capacity (TAC) Assay

The total antioxidant capacity (TAC) of *Tagetes* flower extract samples was measured by using the phosphomolybdate reagent method. In this method, TAC dye was prepared by measuring an equal proportion of 0.6 M sulfuric acid (H_2_SO_4_), 28 mM trisodium phosphate (Na_3_PO_4_), and 4 mM ammonium molybdate. The reaction mixture was prepared by measuring 40 μL of sample extract with 260 μL of TAC dye into wells of the microplate and incubated for 90 min at 95°C. Total antioxidant capacity was determined by measuring absorbance at 695 nm in a microplate reader. The standard curve based on ascorbic acid (0–200 μg/mL) was constructed, and results were expressed as the mass of ascorbic acid equivalent per gram (mg AEE/g) dry weight of *Tagetes* powder sample.

### 2.5 FT‐IR Spectroscopy

The FT‐IR spectroscopy analysis was carried out for structural analysis of freeze‐dried extract samples prepared by UAE by using an FT‐IR spectrometer (PerkinElmer, USA). Each sample was measured (5 mg), pressed on diamond crystal, and transmittance was recorded in the wavelength range of 4000–400 cm^−1^ with a resolution of 4 cm^−1^ at room temperature. The scan rate was set to 16 scans/min for each sample with air as background. Further, curve smoothing and baseline correction were done through Savitzky–Golay and Gaussian fitting functions, respectively, by using Origin 8.6 (OriginLab Inc., MA, USA).

### 2.6 Characterization of *Tagetes* Phenolic Compounds by LC‐ESI‐QTOF‐MS/ MS Analysis

Phenolic compounds of *Tagetes* extract samples were characterized using the advanced analytical technique of Accurate‐Mass Quadrupole Time‐of‐Flight (Q‐TOF) LC/MS, following the previously described method of LC‐ESI‐QTOF‐MS/MS (Shi et al. 2024). The separation was performed using a Synergi Hydro‐RP 80 Å LC column (250 × 4.6 mm, 4 μm) (Phenomenex, Torrance, CA, 202 USA) at 25°C, with a sample injection volume of 20 μL and temperature at 10°C. Buffers and mobile phases were: A [100% Milli‐Q water + 0.1% formic acid] and B [Acetonitrile: Milli‐Q water: formic acid (95:5:0.1)]. Additionally, parameters such as N_2_ gas pressure, gas flow, gradient, and solvent flow were adhered to our research method published by Suleria et al. (2020). In this procedure, 6 μL of filtered (0.45 μm) plant extract samples were analyzed using an Agilent HPLC (1200) coupled with an Agilent Accurate‐Mass Q‐TOF LC/MS (Agilent Technologies, Santa Clara, CA, USA), employing both positive and negative ionization modes to find the highest peaks. Subsequent data analysis was conducted utilizing MS/MS for collection via LC‐ESI‐QTOF‐MS/MS Mass Hunter workstation software (Qualitative Analysis, version B.03.01, Agilent Technologies, Santa Clara, CA, USA).

### 2.7 Quantification of *Tagetes* Bioactive Phenolic Compounds Through HPLC‐DAD Analysis

For quantification of bioactive compounds, *Tagetes* sample extracts (1 mL) prepared with ultrasonication were filtered (0.45 syringe filters), diluted with extraction solvent (1:10), and filled in HPLC vials (Shi et al. 2023). Quantification of targeted bioactive compounds of extract samples was carried out by following the standard method of Suleria et al. (2020). High‐performance liquid chromatography (HPLC, Waters Alliance system, 2690) equipped with a reversed phase column (Synergi Hydro‐RP) of dimensions 4.6 × 250 mm with particle size 4 μm protected by guard column C18 ODS (Phenomenex, Torrance, CA) and connected with a diode array detector (DAD Model 2998, Waters). The binary mobile phase system consists of two mobile phases: Mobile Phase A [Milli‐Q H_2_O/acetonitrile (95:5)] and Mobile Phase B [Milli‐Q H2O/acetonitrile (50/50)]. Flow rate was set to 0.8 mL/min, and the injection volume was set to 25 μL, and the column was operated at room temperature 25°C. Detector wavelengths were set at 280, 320, 370, and 450 for data acquisition. Various compound identification was achieved by comparing retention time (RT) and the absorption spectra of the peak with standards (Sigma‐Aldrich, Castle Hill, NSW, Australia).

### 2.8 Statistical Analysis

All experiments were performed in triplicate by using a randomized complete block design (RCBD). Solvents and marigold flowering stages were considered distinct factors, whereas the parameters evaluated were treated as dependent factors. Analysis of variance (ANOVA) and mean comparison by Tukey's honestly significant differences (HSD) were performed at *p* ≤ 0.05 using the SPSS program (Package version 28.0.1, 2021). Mean values are presented as standard deviation (± SD). Pearson's correlation coefficient at *p* ≤ 0.05 was applied to determine the correlation among various variables, and principal component analysis (PCA) was performed to understand variability using the XLSTAT 2023 package, and the spectrum was visualized by using Origin 8.6 (OriginLab Inc. MA, USA).

## 3 Results and Discussion

### 3.1 Phenolic Content Estimation of *Tagetes* Flower Extracts

Bioactive compounds, especially polyphenolics (phenolics, flavonoids, and tannins) and carotenoids (carotenes, xanthophylls), are commonly found in various parts of medicinal plants, including leaves, flowers, buds, and seeds, etc. *Tagetes erecta* L. is an indigenous plant rich in phytochemicals with potential health benefits. Numerous pieces of evidence report that polyphenolic compounds have attracted great interest from producers and consumers toward functional foods or nutraceuticals owing to their protective role against various age‐related disorders (Caleja et al. 2017; Goudjil et al. 2024).

This study focused on full‐bloom flower samples of the *Tagetes erecta* L. plant to investigate its phenolic and antioxidant properties by varying multiple conditions: drying methods (oven‐dried and freeze‐dried), solvents (ethanol, acetone, hexane), and extraction methods (CSE, UAE) used for extraction to confirm the impact of varying treatments on bioactive compounds. Results showed the total phenolics, flavonoids, and condensed tannins in conventional solvent and ultrasound‐assisted extracted samples. Total phenolic content (TPC) among various treatments showed significant differences (*p* < 0.05) and revealed that the highest phenolic content was observed in ultrasound‐extracted samples (Table 2). The phenolic content in UAE extracts ranged from 0.20 to 74.57 mg GAE/g, whereas in CSE it ranged from 0.14 to 55.35 mg GAE/g. Among solvents, ethanol resulted in higher TPC values, followed by acetone and hexane in both treatments, and showed a consistent trend for both (oven and freeze‐dried) samples. Thus, the highest TPC value was observed for MFEU (74.57 mg GAE/g), followed by MFAU (70.19 mg GAE/g), the freeze‐dried ultrasound‐extracted samples, while the lowest amounts were observed in hexane‐extracted samples [MFHU (0.20 mg GAE/g) > MOHC (0.14 mg GAE/g)]. In this study, total phenolic content was measured by using the Folin–Ciocalteu reagent (FCR), which has the ability to react with phenolic and non‐phenolic compounds (Escarpa and González 2001). As marigold is also rich in other bioactive compounds, including carotenoids, this resulted in higher phenolic content in our study than reported previously (Siriamornpun et al. 2012).

**TABLE 2:** Measurement of total phenolic content, total flavonoid content, and total condensed tannins in freeze‐dried and oven‐dried *Tagetes* flower samples.

Samples		TPC (mg GAE/g)	TFC (mg QE/g)	TCT (mg CE/g)
Conventional Solvent Extraction	MFEC	55.35 ± 1.28^c^	4.36 ± 0.27^d^	—
	MOEC	7.15 ± 0.38^f^	0.94 ± 0.04^f^	—
	MFAC	17.34 ± 1.12^d^	5.23 ± 0.22^c^	—
	MOAC	8.52 ± 0.47^ef^	1.8 ± 0.09^e^	—
	MFHC	0.14 ± 0.07^g^	0.78 ± 0.01^fg^	—
	MOHC	0.17 ± 0.09^g^	0.58 ± 0.02^fg^	67.86 ± 1.40^a^
Ultrasound Assisted Extraction	MFEU	74.57 ± 1.60^a^	8.91 ± 0.25^a^	—
	MOEU	9.39 ± 0.40^e^	0.94 ± 0.04^f^	—
	MFAU	70.19 ± 0.13^b^	6.87 ± 0.42^b^	—
	MOAU	9.85 ± 0.08^e^	1.05 ± 0.02^f^	—
	MFHU	0.20 ± 0.08^g^	0.37 ± 0.01^gh^	—
	MOHU	0.20 ± 0.01^g^	0.01 ± 0.01^h^	33.97 ± 0.35^b^

*Note:* The data shown in the table is mean ± standard deviation (*n* = 3); Lettering (a, b, c, d, e, f, g, h, i) indicates the significant difference in the means (*p* < 0.05) using a one‐way analysis of variance (ANOVA) and Tukey's HSD test. Abbreviations: CE, catechin equivalents; DW, dry weight; GAE, gallic acid equivalents; QE, quercetin equivalents; TCT, total condensed tannins; TFC, total flavonoid contents; TPC, total phenolic contents. Sample abbreviations are explained at Table 1.

Current study results are supported by a previous study by Subbiah et al. that found ultrasonication extraction resulted in higher total phenolic (TPC) values as compared to conventional solvent extracts (Subbiah, Ebrahimi, et al. 2023). The higher phenolic content in UAE samples might be a result of the ultrasonication process that has the power to break the cell wall of plant material and facilitate the extraction of phenolic compounds into extracts (He et al. 2024). The findings of the current research are also supported by previous research results (Tungmunnithum et al. 2020), which reported that the combination of the ultrasound technique with extraction resulted in enriched bioactive components in extracts from medicinal plants that can be utilized in functional food development. These results are also supported by previous evidence that ultrasound radiations cause cavitation in cells to free bound components, making them available for improved extraction (Jalali‐Jivan et al. 2019). Results are also in line with the previous study that reported ethanol as the most efficient solvent due to its polarity for the extraction of phenolics and carotenoids (Li et al. 2007), especially when combined with ultrasonication (Shi et al. 2024). Another study investigated various drying methods and estimated the phenolics and antioxidant activities and found that the freeze‐dried samples consistently resulted in higher phenolic contents, whereas oven‐dried samples resulted in low phenolic content (Subbiah, Duan, et al. 2023). This might be the reason that heating may cause the degradation of phytochemicals and polyphenol oxidase that is involved in antioxidant activities (Lim and Murtijaya 2007; Rabeta and Vithyia 2013).

Flavonoids are one of the predominant classes of polyphenolics found abundantly in almost all medical plants (Roy et al. 2022). These compounds possess strong biological activities including free radical scavenging activities resulting in higher antioxidant potential (Wu et al. 2023). Total flavonoid content (TFC) was measured by the aluminum chloride colorimetric method as AlCl_3_ reacts with the carbonyl group of flavonoids and forms stable complexes (Shraim et al. 2021). Results showed that the highest flavonoid content was observed in ultrasound‐assisted extracts of freeze‐dried flower samples; MFEU (8.91 mg QE/g) followed by MFAU (6.87 mg QE/g). In our study, total tannin content (TTC) was only observed in oven‐dried marigold flower samples. The highest TTC value was 67.86 mg CE/g (MOHC) followed by 33.97 mg CE/g (MOHU). However, surprisingly, the tannin content was not detected in all other solvent extracts other than hexane and in both (UAE, CSE) extraction methods. Previous research also reported no tannin content in marigold flower dried extracts while leave extracts showed tannin content in a few studies (Rajvanshi and Dwivedi 2017). Results are supported by a previous study that followed the same method of extraction for tannins and found the highest tannin content from conventional extraction among all samples and methods (Subbiah, Ebrahimi, et al. 2023). Current study results for TFC are in line with the previous study that reported that the total flavonoid content was significantly higher in fresh and freeze‐dried marigold flower samples as compared to hot air oven‐dried samples (Siriamornpun et al. 2012). Results are also supported by previous research that found TFC values higher for ethanol and acetone extracts in ultra‐sonification samples (Subbiah, Ebrahimi, et al. 2023). The freeze‐dried marigold samples resulted in a higher TFC value due to the release of non‐polar cellular compounds from the inner plant matrix (Pérez‐Gregorio et al. 2011). Previously, higher total flavonoid contents (110–140 mg QE/g) were reported in *Tagetes erecta* L. than in our study findings. This may be a result of differences in extraction methods (supercritical fluid extraction, SCFA) and drying techniques (FIR) used for the extraction of bioactive compounds in their study (Siriamornpun et al. 2012). Evidence reported that the total flavonoid content varies under various extraction methods and conditions including solvent type, concentration, purity, extraction time, temperature, and solute to solvent ratio etc. (Zerajić et al. 2019; Spigno et al. 2007). The phenolic content and compounds also differ among various species based on different regions, climate conditions, and cultivation methods (Suleria et al. 2020). Evidence also reported that freeze drying can preserve phenolics (TPC, TFC, and TCT) and improve the release of bound phenolics and protect phenolic compounds through enzyme degradation (oxidative, hydrolytic) (Ballesteros et al. 2017; Chang et al. 2006).

### 3.2 Determination of Antioxidant Potential of *Tagetes* Flower Extracts

The antioxidant potential of *Tagetes* full‐bloom flower samples was further investigated by employing various antioxidant assay methods based on different mechanisms. In this study, seven different antioxidant assays, including DPPH, FRAP, ABTS, RPA, ^•^OH‐RSA, FICA, and TAC, were performed to evaluate the radical scavenging activities (RSA), metal chelating, hydrogen atom donating, and reducing potential of marigold full‐bloom flower (MFP) extracts. Antioxidant compounds protect the human body owing to their ability to reduce oxidative stress by scavenging free radicals, binding the metals, and supplying hydrogen atoms to the reactive system (Kıran et al. 2023). *Tagetes* flowers are a rich source of bioactive compounds, including phenolics, flavonoids, and carotenoids, including lutein, imparting antioxidant activities, making it a complex system. Thus, using a combination of antioxidant and radical scavenging assays provides a more comprehensive understanding of antioxidant activities (Lutz et al. 2015). Results for all antioxidant assays were recorded in mg standard equivalent per gram and analyzed by Tukey statistical (*p* < 0.05) test to compare the effect of various drying and extraction methods on the antioxidant potential of MFP extracts (Table 3).

**TABLE 3:** Determination of antioxidant activities in marigold plant extract samples.

Samples		DPPH	FRAP	ABTS	TAC	OH^−^	FICA	RPA
		(mg AAE/g)	(mg AAE/g)	(mg AAE/g)	(mg AAE/g)	(mg TE/g)	(mg EE/g)	(mg TE/g)
Conventional Solvent Extraction (CSE)	MFEC	18.43 ± 0.01^a^	60.33 ± 1.35^a^	31.63 ± 0.55^a^	58.26 ± 0.37^b^	252.18 ± 1.07^c^	2.00 ± 0.15^ab^	38.86 ± 0.31^d^
	MOEC	14.90 ± 0.51^d^	15.57 ± 0.15^d^	10.90 ± 0.08^d^	14.21 ± 0.33^d^	25.45 ± 1.43^de^	1.59 ± 0.03^d^	32.06 ± 0.95^e^
	MFAC	17.36 ± 0.29^b^	59.10 ± 1.05^a^	30.16 ± 011^b^	59.12 ± 2.21^b^	252.6 ± 0.55^c^	1.86 ± 0.05^bc^	52.89 ± 1.18^c^
	MOAC	15.00 ± 0.28^d^	17.65 ± 0.79^c^	8.89 ± 0.15^e^	9.38 ± 0.2^e^	18.59 ± 0.72^f^	1.74 ± 0.07^cd^	33.71 ± 0.65^e^
	MFHC	1.36 ± 0.07^f^	0.74 ± 0.03^e^	2.61 ± 0.14^f^	5.49 ± 0.09^f^	1.29 ± 0.05^g^	0.78 ± 0.02^g^	0.23 ± 0.01^g^
	MOHC	1.02 ± 0.03^f^	0.36 ± 0.01^e^	1.99 ± 0.01^fg^	4.25 ± 0.14^fg^	1.21 ± 0.06^g^	1.01 ± 0.03^e^	0.08 ± 0.03^g^
Ultrasound Assisted Extraction (UAE)	MFEU	18.49 ± 0.07^a^	51.87 ± 0.63^b^	32.14 ± 0.65^a^	70.25 ± 0.77^a^	261.24 ± 1.5^b^	2.14 ± 0.01^a^	66.39 ± 1.51^a^
	MOEU	15.81 ± 0.24^c^	14.75 ± 0.67^d^	10.94 ± 0.02^d^	13.20 ± 0.49^d^	25.87 ± 0.62^d^	1.77 ± 0.05^cd^	41.06 ± 1.65^d^
	MFAU	17.14 ± 0.11^b^	59.76 ± 0.73^a^	27.17 ± 0.7^c^	54.95 ± 0.16^c^	267.33 ± 1.37^a^	2.17 ± 0.03 ^a^	57.83 ± 1.61^b^
	MOAU	12.78 ± 0.29^e^	16.28 ± 0.56^cd^	8.88 ± 0.01^e^	1.84 ± 0.01^h^	23.11 ± 0.25^e^	1.94 ± 0.07^b^	28.01 ± 1.52^f^
	MFHU	0.349 ± 0.01^g^	0.42 ± 0.02^e^	1.63 ± 0.04^g^	4.69 ± 0.12^f^	2.02 ± 0.07^g^	0.95 ± 0.01^ef^	0.60 ± 0.03^g^
	MOHU	0.98 ± 0.02^fg^	0.30 ± 0.01^e^	1.53 ± 0.01^g^	2.29 ± 0.05^gh^	1.82 ± 0.11^g^	0.85 ± 0.05^ef^	0.43 ± 0.01^g^

*Note:* The data shown in the table as mean ± standard deviation (*n* = 3); lettering (a, b, c, d, e, f, g) indicates the significant differences in the means (*p* < 0.05) using a one‐way analysis of variance (ANOVA) and Tukey's HSD test. Abbreviations: ^•^OH‐RSA, hydroxyl‐radical scavenging activity; AAE, ascorbic acid equivalents; ABTS, 2,2′‐azino‐bis‐3‐ethylbenzothiazoline‐6‐sulfonic acid assay; DPPH, 2,2′‐diphenyl‐1‐picrylhydrazyl assay; EE, EDTA (ethylenediaminetetraacetic acid) equivalent; FICA, ferrous ion chelating activity; FRAP, ferric reducing antioxidant power assay; RPA, reducing power assay; TAC, total antioxidant capacity. Samples' abbreviations are explained at Table 1.

The DPPH (2,2′‐diphenyl‐1‐picrylhydrazyl) assay is widely recognized as an easy and effective method to assess the antioxidant potential in medicinal plant extracts. It is widely performed to evaluate the radical scavenging potential attributed to polyphenolic compounds (Yang et al. 2020). DPPH works on a scavenging mechanism in which 2,2′‐diphenyl‐1‐picrylhydrazyl forms a stable radical by donating a proton, indicated by a color change (turned violet color into light yellow) (Szabo et al. 2007). Results showed that the highest DPPH value was observed in freeze‐dried powder extract in ethanol (MFEU: 18.49 mg AAE/g), followed by its acetone extracts (MFAU: 17.14 mg AAE/g) prepared by UAE method. Overall results showed that ultra‐sonified freeze‐dried (MFU) extract samples resulted in higher DPPH potential than the oven‐dried solvent extracts (MOC) of marigold full‐bloom flowers.

FRAP assay is widely performed to measure the reducing power of extract samples due to its ability to donate electrons. It reduces ferric (Fe^+3^) TPTZ complex to ferrous (Fe^+2^) TPTZ complex ions reflected through the color change of sample (colorless➔blue color). Table 3 summarizes the reducing potential of the oven and freeze‐dried full‐bloom flower sample extracts of marigold, which were extracted via CSE and UAE methods. Results showed that in CS extracts, the highest FRAP value was observed with ethanol in MFEC (60.33 mg AAE/g) followed by acetone extract MFAC (59.10 mg AAE/g) of freeze‐dried *Tagetes* samples. While in ultrasound‐assisted extracts, the highest FRAP was observed with acetone in MFAU (59.76 mg AAE/g) followed by ethanol extract MFEU (51.87 mg AAE/g) of freeze‐dried marigold flower samples. However, the oven‐dried extract samples of *Tagetes* extracts resulted in significantly lower values than the freeze‐dried samples for both conventional and ultrasound extracts (UAE > CSE). The current study trend was supported by previous research reporting that oven‐dried sample extracts resulted in lower antioxidant potential. This might be due to the slow heat transfer mechanism in the oven drying process, which caused insufficient denaturation of polyphenolic compounds (polyphenol oxidase) involved in the degradation of antioxidant compounds (Rabeta and Vithyia 2013).

The ABTS assay is similar to FRAP (ferric reducing assay power) based on their ability to donate hydrogen atoms. It measures the scavenging potential of antioxidant compounds reflected by color change (turning mixture to colorless) on scavenging free radical cations (ABTS^+^) to form stable radicals (Dasgupta and Klein 2014). The highest ABTS potential was observed in MFEU (32.14 mg AAE/g), ultrasound‐assisted extracts prepared with ethanol followed by its acetone extracts; MFAU (27.17 mg AAE/g). Current study results showed that the extraction methods resulted in non‐significant differences, but the drying methods resulted in significantly higher differences in *Tagetes* extract samples. In CSE, the oven‐dried samples of marigold flowers showed a lower ABTS value for MOEC (10.90 mg AAE/g), while the freeze‐dried sample of the same solvent extract resulted in a higher ABTS value MFEC (31.63 mg AAE/g). The current study trend for ABTS results is in line with the previous study investigating different drying methods' effect on antioxidant activities and reporting that the ABTS potential was observed to be higher in freeze‐dried samples than the oven‐dried sample extracts. This might be the reason that heating may cause the degradation of phytochemicals and polyphenol oxidase that are involved in antioxidant activities, while in freeze drying, phenolic compounds get more concentrated on ice trap (Subbiah, Duan, et al. 2023).

In this phase of the study, the bloom *Tagetes* samples were fully evaluated by employing other antioxidant assays including FICA, RPA, and OH^−^ RSA assay. These assays were performed to evaluate the radical scavenging activities (RSA) attributed to bioactive compounds of marigold flower samples. Hydroxyl radical scavenging (^•^OH^−^ RSA) assay was performed to assess the ability of marigold flower samples to scavenge hydroxyl free radicals that may cause oxidative stress to the human body (Kutlu et al. 2014). Study results showed that the highest OH^−^ RSA was observed in ultrasonicated freeze‐dried MFFP extract MFAU (267.33 mg TE/g, 70% acetone), followed by MFEU (261.24 mg TE/g, 70% ethanol). In conventional solvent extracts, the highest OH^−^ RSA was observed in freeze‐dried sample extracts MFAC: 252.60 mg TE/g > MFEC: 252.18 mg TE/g, while the lowest was observed for MOHC: 1.21 mg TE/g.

Ferrous ion chelating activity assay (FICA) was performed to assess the metal chelating ability of marigold full‐bloom flower samples reflected by the conversion of ferrozine to ferrous ions (Gulcin and Alwasel 2022). Study results showed that among the extractions, UAE showed the highest antioxidant activities for MFAU (2.17 mg EDTA/g) > MFEU (2.14 mg EDTA/g). Assay results showed that the freeze‐dried samples resulted in higher FICA than oven‐dried samples; MFEC (2.00 mg EDTA/g) > MOEC (1.56 mg EDTA/g) for all sample extracts except for MOHC (1.01 mg EDTA/g) > MFHC (0.78 mg EDTA/g).

Reducing power assay (RPA) showed a similar trend with other radical scavenging assays, resulting in higher antioxidant potential for ultrasonication extract samples of *Tagetes* than the conventional solvent extracts. The highest RPA was observed for MFEU (66.39 mg TE/g) > MFAU (57.83 mg TE/g) > MFAC (52.89 mg TE/g) > MFEC (38.86 mg TE/g). Overall, among the methods, the freeze‐drying and ultrasonication methods resulted in higher antioxidant activities, while among the solvents tested, 70% ethanol and 70% acetone exhibited higher antioxidant activities in all sample treatments.

The total antioxidant assay is one of the common assays employed as an indicator of the antioxidant activities of food materials. It is the ability of bioactive polyphenols to neutralize the free radicals that can cause oxidative stress or damage to the system. The total antioxidant potential of bioactive/polyphenolic compounds was measured by the TAC assay based on the electron transfer mechanism caused by the reduction of molybdenum (VI) to molybdenum (V) (Suleria et al. 2020). The TAC assay was performed to investigate the effect of different drying methods and extraction techniques by employing various solvents to assess the antioxidant potential attributed to bioactive compounds including phenolics, flavonoids, and carotenoids present in extract samples of marigold full bloom flowers. Study results showed that the trend for TAC values for drying methods was observed to be higher in freeze‐dried samples followed by oven‐dried samples. The highest TAC value was observed in MFEU (70.25 mg AAE/g) followed by MFAC (59.12 mg AAE/g) > MFEC (58.26 mg AAE/g) > MFAU (54.94 mg AAE/g). The total antioxidant activities of marigold freeze‐dried powder (MFP) resulted in higher TAC values, revealing that the drying method significantly affects total antioxidant potential more than the choice of solvents. Our study objective results were also in line with the previous study that reported freeze drying as one of the most efficient drying methods compared to oven and vacuum drying methods for polyphenolic compounds and resulted in antioxidant potential at 40°C. Evidence reported that the heat involved in other drying methods (oven + vacuum) would cause significant losses of antioxidant compounds due to their unstable nature and is considered the responsible factor for the loss of antioxidant activities (Bhattacharyya et al. 2010; Subbiah, Duan, et al. 2023). The significant differences in TAC values also revealed that along with drying, extraction methods and solvents also caused significant (*p <* 0.05) differences. Evidence supported by previous research evaluated various extraction methods and solvents and reported that the ultrasonication extracts and organic solvents, ethanol and acetone, resulted in higher antioxidant potential than conventional extraction methods (Subbiah, Ebrahimi, et al. 2023). Conclusively, the study found that freeze‐drying prior to ultrasonication is a more efficient method to extract bioactive phenolic compounds from *Tagetes* flowers with potential antioxidant activities when extracted with 70% ethanol and 70% acetone being the recommended solvents for the extraction of polyphenolic antioxidant compounds.

### 3.3 Correlation Between Phenolic Content and Antioxidant Assays

**FIGURE 1:**
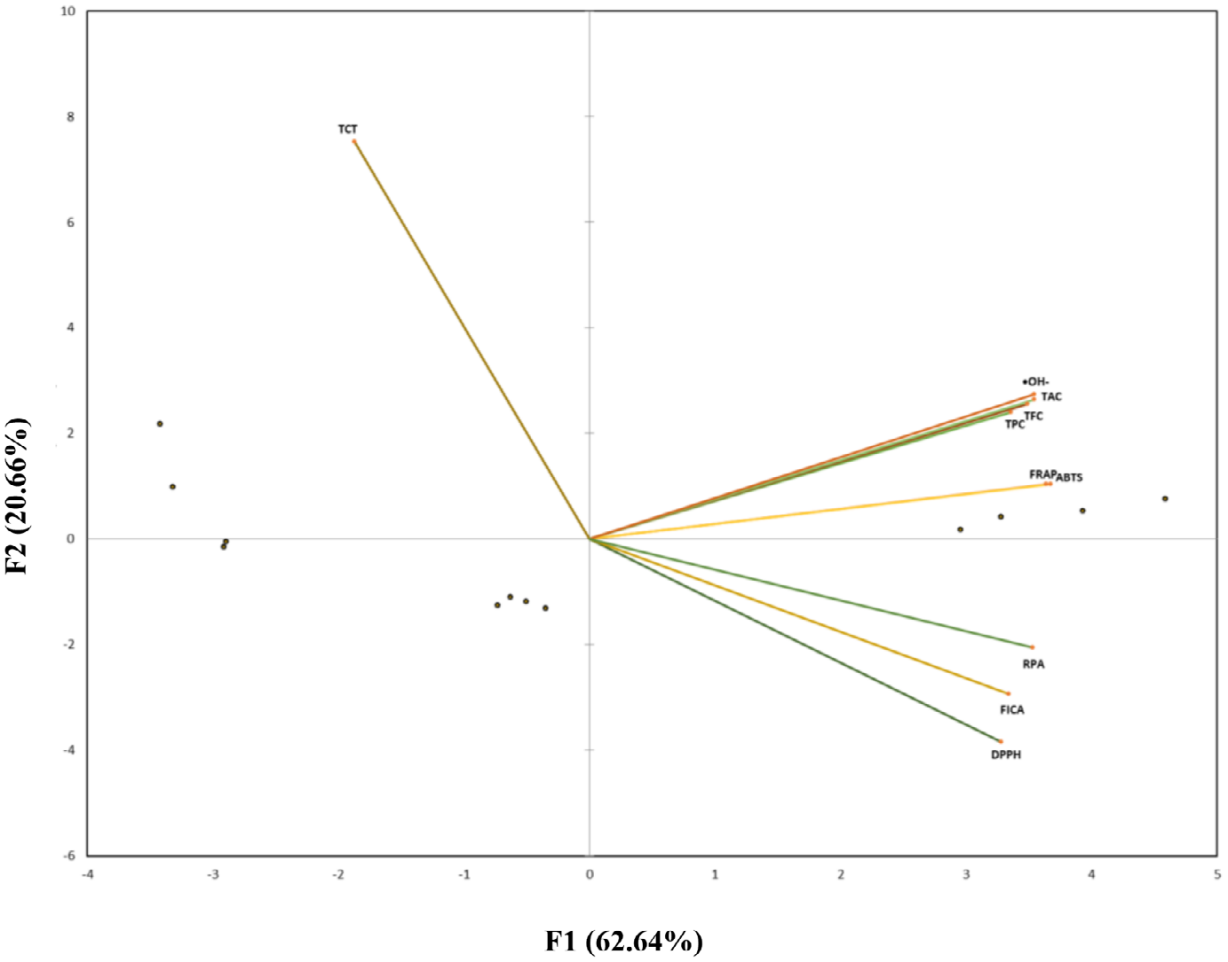
Principal component analysis (PCA) of the phenolic content (TPC, TFC, and TCT) and antioxidant activities (DPPH, FRAP, ABTS, ^•^OH^−^, TAC, RPA, and FICA).

The correlation between phenolic content (TPC, TFC, and TCT) and antioxidant assays (DPPH, FRAP, ABTS, ^•^OH^−^, RPA, FICA, and TAC) was performed with a Pearson's correlation test (Table 4). Additionally, principal component analysis (PCA) was performed to investigate the relationship between phenolic contents and antioxidant potential of *Tegetes* extracts prepared by ultra‐sonified and conventional methods (Figure 1).

**TABLE 4:** Pearson correlation coefficients (R) between phenolic content (TPC, TFC, TCT) and antioxidant activities (DPPH, FRAP, ABTS, •OH^−^, TAC, RPA, and FICA).

Variable	TPC	TFC	TCT	DPPH	FRAP	ABTS	TAC	•OH‐	FICA
TFC	0.924**								
TCT	−0.323	−0.335							
DPPH	0.664*	0.681*	−0.575						
FRAP	0.851**	0.893**	−0.421	0.815*					
ABTS	0.863**	0.913**	−0.426	0.829*	0.986**				
TAC	0.88**	0.949**	−0.345	0.711*	0.955**	0.976**			
•OH—	0.885**	0.936**	−0.335	0.699*	0.977**	0.973**	0.985**		
FICA	0.744*	0.733*	−0.51	0.958**	0.828**	0.827**	0.712*	0.729*	
RPA	0.778*	0.846**	−0.53	0.944**	0.866**	0.885**	0.822*	0.805*	0.939*

* Significant correlation at *p* ≤ 0.05.

** Significant correlation at *p* ≤ 0.01.

A total variability of 83.30% from initial data was explained by the first two factors i.e., active variables F1 and F2 (Figure 1). Among antioxidant assays, DPPH, FRAP, ABTS, TAC, OH‐, FICA, and RPA are positively and strongly correlated with phenolic content (TPC and TFC) (*p* ≤ 0.01), while a negative and non‐significant relationship was observed with TCT except for DPPH, which has a significant negative association.

**FIGURE 2:**
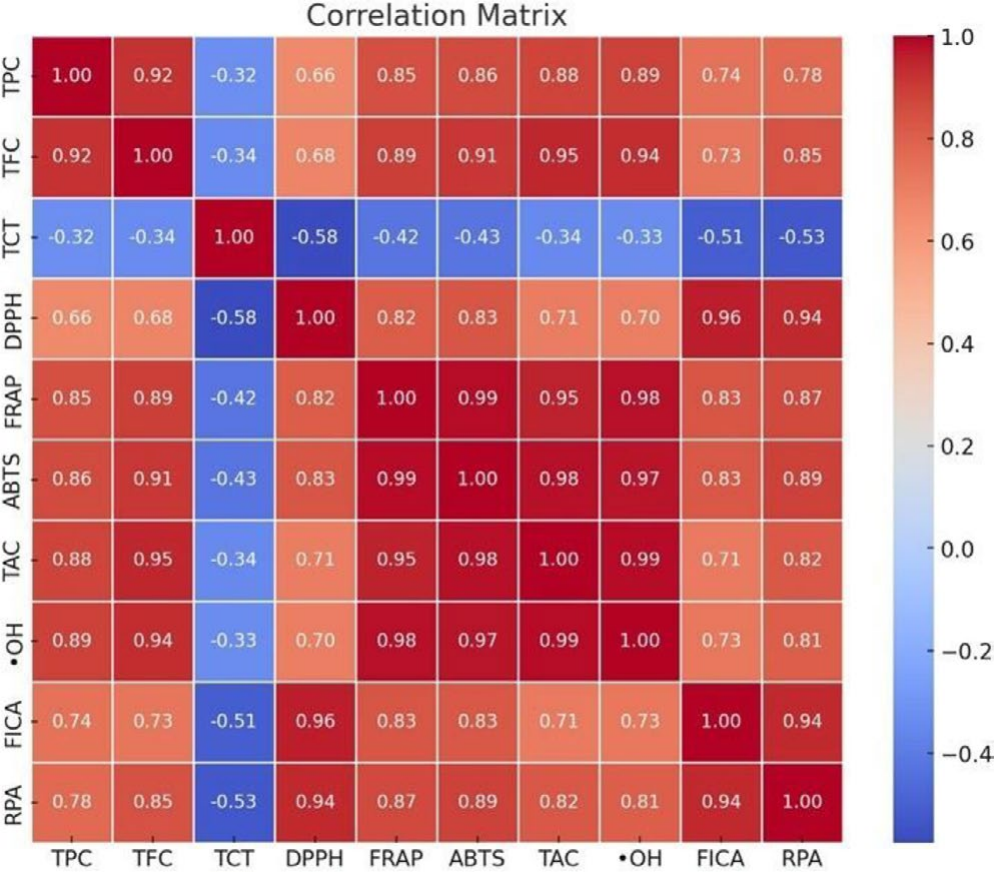
Correlation coefficients (*r*) between phenolic content (TPC, TFC, and TCT) and antioxidant activities (DPPH, FRAP, ABTS, •OH^−^, TAC, RPA, and FICA).

The study results are supported by previous research that reported a highly significant positive correlation between antioxidant assays and phenolic contents, indicating that phenolic and flavonoid compounds present in medicinal plants exhibit strong radical scavenging activities (Figure 2). This antioxidant potential is attributed to the number and position of functional groups [methoxy (‐OCH_3_), hydroxy (‐OH)] attached to the aromatic ring structure of phenolic acids. This potential is increased with an increase in functional groups, meaning the higher the methoxy (‐OCH_3_) and hydroxy (‐OH) groups, the greater the antioxidant activities. Total phenolic content (TPC) and total flavonoid content (TFC) were significantly and positively correlated with antioxidant assays, including DPPH (*r* = 0.66, 0.68), FRAP (*r* = 0.85, 0.89), ABTS (*r* = 0.86, 0.91), TAC (*r* = 0.88, 0.95), OH‐ (*r* = 0.89, 0.94), FICA (*r* = 0.74, 0.73), and RPA (*r* = 0.78, 0.85) (*p* ≤ 0.01). In addition, TCT was negatively and significantly correlated only with DPPH (*r* = −0.58) and was non‐significantly associated with remaining antioxidants. Study findings suggested that the antioxidant activities are mostly contributed by TPC and TFC content exhibited by *Tagetes* species (Akshaya et al. 2017). The significant correlation exhibited by TPC and TFC with antioxidant assays agreed with a previous study investigating bioactive compounds present in *Tagetes* and their correlation with radical scavenging activities (Youssef et al. 2020; Siddiqa et al. 2025).

### 3.4 FT‐IR Spectroscopy

**FIGURE 3:**
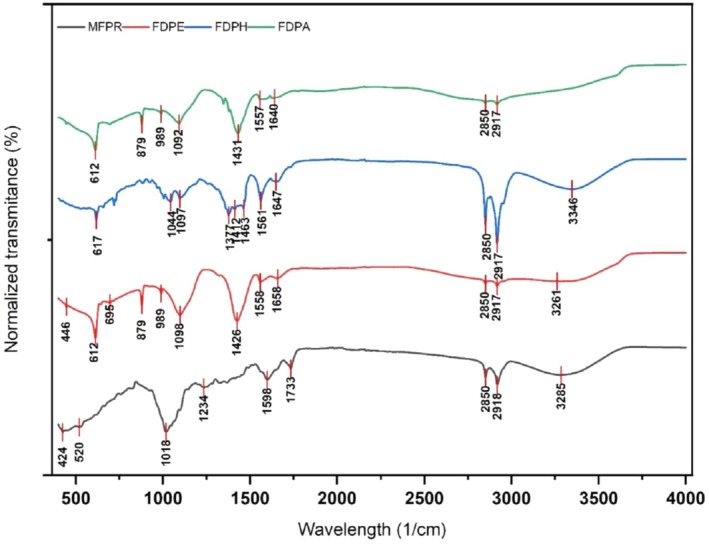
FT‐IR spectra of MFPs.

The FT‐IT spectra of various freeze‐dried purified extracts of *Tagetes* were shown in Figure 3. FT‐IR spectrum showing the impact of extracting solvents and treatment on functional groups of purified oleoresins in comparison to raw fine powder (MFPR) showed prominent peaks at 3285, 2918, and 2850 cm^−1^ which corresponded to stretching vibrations of O–H and C–H stretches (Zhao et al. 2020). Stretching peaks at 2917 and 2850 cm^−1^ were observed in all FD extract samples; however, 3261 [MDPE] > 3346 cm^−1^ [MDPH] showed transmittance due to O–H, ‐C–H, and =C–H stretching vibrations in UAE‐FD extracts of ethanol (Manzoor et al. 2022). Peaks observed in the double bonds stretching region (1800–1500 cm^−1^) showed transmission at 1647 [FDPH], 1658 [FDPE], and 1640 [FDPA] due to the presence of C=O bonds of ester groups from flavonoids and carotenoids. Similar peaks were identified in extract samples with increased transmittance (%) possibly due to the conversion of functional groups resulted through the UAE process. Results are supported by a previous study that reported similar groups in specific percentage transmittance reflecting non‐significant effects on the functionality of bioactive compounds (Manzoor et al. 2019).

### 3.5 LC‐ESI‐QToF MS/MS Characterization of Phenolic Compounds of *Tagetes* Flower Extracts

The untargeted identification and characterization of bioactive polyphenolic compounds of various *Tagetes* samples were performed through LC‐ESI‐QTOF. LCMS analysis was performed by using mass spectra (*m/z*) values of Agilent LC–MS Qualitative software by taking both positive [M + H]^+^ and negative [M‐H]^−^ ionization modes with mass error less than ±5 ppm. Further compound characterization and verification were performed by MS/MS analysis and *m/z* identification in relation to Personal Compound Database Library (PCDL library score > 80). The present study tentatively identified 33 polyphenolic compounds including phenolic acids (13), carotenoids (3), flavonoids (14), and other polyphenols (3) based on their retention time, mol. weight, and m/z value of fragment ions in different extract samples. With growing consumer knowledge and interest in the antioxidant potential and health benefits of bioactive compounds, medicinal and unconventional edible plants have attracted significant attention from the pharmaceutical and nutraceutical sectors (Lescano et al. 2019; Kiani et al. 2023).

#### 3.5.1 Phenolic Acids

Polyphenols are the most abundant bioactive compounds present in various parts of medicinal plants and are recognized for their promising health benefits, including antioxidant and anti‐inflammatory activities (Shi et al. 2022). A total of thirteen phenolic acids (13) were tentatively identified and confirmed by mass fragmentation pattern. Identified phenolic acids are categorized as hydroxybenzoic acids (6) and hydroxycinnamic acids (7). Among hydroxybenzoic acids, Compound 1: gallic acid (*m/z* 169.0137), Compound 2: gallic acid 4‐*O‐*glucoside (m/z 331.0671), and Compound 3: 3,4‐*O*‐Dimethylgallic acid (*m/z* 197.0435) were identified in negative mode of ionization in various extract samples of MFEU, MFAU, MFEC, and MFHU (Table 5). Further, MS/MS analysis confirmed the fragment ions of Compound 1 [*m/z* 125] and Compound 3 [*m/z* 125] resulted from neutral loss of CO_2_ (44 Da) from precursor ions, while Compound 2 [*m/z* 169, 125] product ions resulted through characteristic loss of hexosyl moiety (glucoside 162 Da) and CO_2_ (44 Da), respectively, from parent ions (Szewczyk and Olech 2017). Results are supported by previous studies that found phenolic compounds in various extract samples of medicinal plants, fruits, and seaweeds. Evidence reported that among various compounds, gallic acid is an important phytochemical abundantly found in medicinal plants (Genwali et al. 2013) and recognized for its antioxidant potential against several chronic disorders. Another important metabolite of gallic acid, 3,4‐*O‐*Dimethylgallic acid (Compound 3), is mostly found in various parts of fruits and has potent antioxidant, anticarcinogenic, antimutagenic, and anti‐inflammatory properties (Yücetepe et al. 2021).

**TABLE 5:** Characterization of polyphenolic compounds in *Tagetes* flower samples by LC‐ESI‐QToF‐MS/MS.

No.	Proposed compounds	Molecular formula	RT (min)	Ionization (ESI^+^/ESI^−^)	Molecular weight	Theoretical (*m/z*)	Observed (*m/z*)	Error (ppm)	MS/MS productions	Medicinal samples
**Phenolic acid**										
**Hydroxybenzoic acids**										
1	Gallic acid*	C_7_H_6_O_5_	10.04	[M‐H]^−^	170.0211	169.0138	169.0137	−0.6	125	MFEU, MFAU, MFEC, MFHU
2	Gallic acid 4‐*O*‐glucoside	C_13_H_16_O_10_	15.07	[M‐H]^−^	332.0741	331.0668	331.0671	0.9	169, 125	MFEU, MFAU
3	3,4‐*O*‐Dimethylgallic acid	C_9_H_10_O_5_	14.76	[M‐H]^−^	198.0509	197.0436	197.0435	−0.5	153, 139, 125, 111	MFEU, MFEC
4	Benzoic acid	C_7_H_6_O_2_	14.32	[M+H]^+^	122.0377	123.045	123.0451	0.8	121	MFEU
5	2‐Hydroxybenzoic acid	C_7_H_6_O_3_	13.71	**[M‐H]^−^	138.032	137.0252	137.0256	2.9	93	MFEC, MOEC
6	4‐Hydroxybenzoic acid 4‐*O*‐glucoside	C_13_H_16_O_8_	17.18	[M‐H]^−^	300.0183	299.0110	299.0111	0.3	255, 137	MFAU, MFAC, MFEC
**Hydroxycinnamic acids**										
7	*m*‐Coumaric acid*	C_9_H_8_O_3_	14.31	**[M+H]^+^	164.0472	165.0545	165.054	−3.0	119	MFEU, MFAU
8	Ferulic acid 4‐*O*‐glucoside	C_16_H_20_O_9_	4.573	[M‐H]^−^	356.111	355.1037	355.1044	2.0	193, 178, 149, 134	MFEU, MFAU, MFAC
9	*p*‐Coumaric acid 4‐*O*‐glucoside	C_15_H_18_O_8_	20.27	[M‐H]^−^	326.0712	325.0639	325.0629	−3.1	163	MFEC, MFEU
10	*p*‐Coumaric acid ethyl ester	C_11_H_12_O_3_	19.32	[M‐H]^−^	192.0784	191.0711	191.0711	0.0	143, 133	MFEC, MFEU
11	Ferulic acid*	C_10_H_10_O_4_	4.483	[M‐H]^−^	194.0572	193.0499	193.0493	−3.1	178, 149, 134	MFEU, MFAU, MFEC
12	Ferulic acid 4‐*O*‐glucuronide	C_16_H_18_O_10_	7.087	**[M+H]^+^	370.0886	371.0959	369.0959	−0.0	193.0	MFEC, MFEU
**Carotenoids**										
13	(all‐*E*)‐lutein*	C_40_H_56_O_2_	20.05	[M+H]^+^	568.87117	569.8784	569.8768	−2.7	495, 533, 551	MFEU, MFAU, MFHU
14	(all‐*E*)‐zeaxanthin*	C_40_H_56_O_2_	15.01	[M+H]^+^	568.9970	570.0043	570.0042	−0.2	477, 533, 551	MFEU, MFAU, MFHU, MOHU
15	(all‐*E)*‐violaxanthin‐3‐*O*‐myristate‐3‐O′‐palmitate*	C_40_H_56_O_4_	26.62	**[M‐H]^−^	1049.8521	1048.8448	1048.4398	−0.7	793, 547	MFEU, MFHU, MFAU
**Flavanols**										
16	Quercetin 3‐*O*‐arabinoside	C_20_ H_18_ O_11_	56.10	[M‐H]—	434.0821	433.076	433.077	−0.8	301	MFEC, MFEU
17	(+)‐Gallocatechin	C_15_H_14_O_7_	19.88	[M‐H]^−^	306.0756	305.0651	305.0652	0.2	261, 219	MFAU, MFEU
18	Procyanidin dimer B7	C_30_H_26_O_12_	24.86	[M‐H]^−^	578.142	577.1347	577.1352	0.9	451	MFEU, MFEC, MFAU
**Flavanones**										
19	Neo‐hesperidin	C_28_H_34_O_15_	24.11	**[M+H]^+^	610.1877	611.1951	611.1954	0.7	593, 465, 449, 303	MFEU
20	3‘,4‘,7‐Trihydroxyisoflavanone	C_15_H_12_O_5_	26.69	**[M‐H]^−^	272.0710	271.0637	271.0631	−2.2	177, 151, 119, 107	MFAU, MFEC
21	Luteolin 7*‐O‐(*2‐apiosylglucoside)*	C_26_H_28_O_15_	52.01	[M+H]^+^	580.1714	581.1787	581.1786	−0.2	419, 401, 383	MFEC, MFEU
22	Myricetin 3‐*O*‐arabinoside	C_22_H_18_O_12_	19.59	[M‐H]^−^	474.0783	473.0710	473.0708	−0.4	293, 311	MFAU
**Flavonols**										
23	Kaempferol 3,7‐*O*‐diglucoside*	C_27_H_30_O_16_	24.21	[M‐H]^−^	610.1522	609.1412	609.1411	0.4	285, 447	MFEU, MFAU, MFHU
24	Quercetin 3′‐*O*‐glucuronide	C_21_H_18_O_13_	29.88	[M‐H]^−^	478.0746	477.0673	477.0682	1.9	301	MFEU, MFAU
25	Myricetin 3‐*O*‐galactoside	C_21_H_20_O_13_	21.50	[M‐H]^−^	480.0811	479.0737	479.0735	−0.4	317	MFAU
26	Quercetin 3‐*O*‐xylosylrutinoside	C_32_H_38_O_20_	52.21	**[M‐H]^−^	742.1492	741.1419	741.1418	−0.1	479, 317	MFEC, MFEU
**Isoflavonoids**										
27	2‘,7‐Dihydroxy‐4‘,5‘‐dimethoxyisoflavone	C_17_H_14_O_6_	17.12	[M+H]^+^	314.067	315.0743	315.0741	−1.0	300, 282	MFAU
28	2‐Dehydro‐*O‐*desmethylangolensin	C_15_H_12_O_4_	18.26	[M‐H]^−^	256.0723	255.0650	255.0649	−0.4	135, 119	MFEU, MFAU
29	5,6,7,3‘,4‘‐Pentahydroxyisoflavone	C_15_H_10_O_7_	25.05	[M+H]^+^	302.0437	303.0510	303.0521	3.3	285, 257	MFHU
30	3‘‐Hydroxygenistein	C_15_H_10_O_6_	25.96	[M+H]^+^	286.0505	287.0578	287.0581	−1.0	269, 259	MFEC
**Other polyphenols**										
31	Esculin	C_15_H_16_O_9_	15.98	[M‐H]^−^	340.0321	339.0247	339.0245	−4.0	177	MFHC
32	Tyrosol	C_8_H_10_O_2_	28.68	[M+H]^+^	138.067	139.0743	139.0743	0.0	138	MFHU, MFAC
33	Coumarin*	C_9_H_6_O_2_	3.311	[M+H]^+^	146.0357	147.0431	147.0431	0.0	103, 91	MFAU, MFEU

*Note:* *Compound was detected in more than one samples. **Compounds were detected in both negative [M‐H]^−^ and positive [M+H]^+^ mode of ionization while only single mode data was presented. Samples were mentioned in numbers.

Other phenolic compounds of the hydroxybenzoic acid group were: Compound 4, Benzoic acid identified at RT 14.32 as [*m/z* 123.0451] in positive mode [M + H]^+^ and its derivatives; Compound 5 (2‐hydroxybenzoic acid) [*m/z* 137.0252] and Compound 6 (4‐hydroxybenzoic acid 4‐*O*‐glucoside) [*m/z* 299.0754] were observed in negative mode of ionization [M‐H]^−^ at RT 13.71 and 17.18 (min), respectively. The presence of 2‐hydroxybenzoic acid was confirmed by product ion *m/z* 93 resulting from the removal of CO_2_ (44 Da), and 4‐hydroxybenzoic acid 4‐*O*‐glucoside resulted in fragment ions *m/z* 255, 137 via the loss of CO_2_ (44 Da) and glucoside (162 Da), respectively, from its parent ions *m/z* 299 [M‐H—44, M‐H –162] (Liu et al. 2024).

Hydroxycinnamic acid is also an important subclass of phenolic compounds widely found in various plants (especially Brassica) and fruits (Khalil et al. 2024; Fan et al. 2022). Six hydroxycinnamic compounds including coumaric and ferulic acid and their derivatives were found in various samples, with most samples being ultrasound extracts of freeze‐dried Tagetes samples. Compound 7 observed at RT 14.31 (min) was tentatively identified as *m*‐Coumaric acid with observed mass [*m/z* 165.054], confirmed by product ion [*m/z* 119] at [M‐H]^−^ through the loss of H‐COOH (46 Da) (Suleria et al. 2020). Other derivatives of coumaric acid, *p*‐Coumaric acid (compound 9) and 4‐*O‐*glucoside and *p*‐Coumaric acid ethyl ester (compound 10) were observed in negative mode of ionization [M‐H]^−^ with observed mass [*m/z* 325.0639, *m/z* 191.0711]. Compound 12 was tentatively identified as ferulic acid [*m/z* 193.0493] in negative mode [M‐H]− at [RT 4.48 min]. Resulting compounds were confirmed by MS/MS analysis showing the fragment ions of ferulic acid [*m/z* 178] resulted via sequential loss of CH_3_–15 Da and [*m/z* 149] resulted by removal of CO_2_–44 Da, while the fragment ions [*m/z* 134] resulted from losing CH_3_—CO_2_ [15–44 Da]. Coumaric acid, ferulic acid, and their glucosides are recognized and have been extensively studied for plant‐based drugs owing to their potential therapeutic defensive activities to control tumor proliferation through EGFR down‐regulation, hepatic injuries, and by improving lipid profile (Roy et al. 2016).

#### 3.5.2 Carotenoids

Carotenoid compounds are fat‐soluble pigments naturally biosynthesized in higher plants and some fruits identified by LCMS/MS (Petry and Mercadante 2016). Carotenoids are recognized for their potential to reduce the risk of cancer, cardiovascular, and other age‐related disorders including age‐related macular disorders and cataracts (Duan et al. 2024).

Three important carotenoid compounds were tentatively detected, including lutein, zeaxanthin, and violaxanthin, with observed mass [*m/z* 569.8768, 570.0042] and [*m/z* 1048.4398] in [M + H]^+^ and [M‐H]^−^ mode of ionization. Further compounds identified through product ions m/z 495, 533, and 551 resulted from the loss of C_4_H_6_O_2_ (74 Da) and H_3_O_2_ (36 Da), indicating the cleavage of the hydroxyl group due to the fragmentation of xanthophyll and one molecule of H_2_O (18 Da) (Saini et al. 2023; Hegde et al. 2023).

#### 3.5.3 Flavonoids

Flavonoids are a prevalent group of phenolic compounds detected in various *Tagetes* extract samples. A total of 14 compounds were tentatively identified, including flavanols (3), flavanones (3), flavonols (4), and iso‐flavonoids (4). Compound 17 [Quercetin 3‐*O*‐arabinoside], with observed mass [*m/z* 433.077], detected at RT 56.10 (min) in the negative mode of ionization, was identified by product ion m/z resulted through loss of pentose moiety C_5_H_8_O_4_ (Ali et al. 2021). Compound 22 was identified as Luteolin 7‐*O‐*(2‐apiosylglucoside), with observed mass [*m/z* 581.1786] at RT 52.01 (min) and [M + H]^+^ confirmed by fragment ions *m/z* 419, 401, and 383 in MS/MS mass spectra.

Important flavonol compounds including kaempferol, quercetin, and myricetin conjugates were tentatively identified. Compound 23; Kaempferol 3,7‐*O*‐diglucoside with observed mass [*m/z* 609.1412] was detected at RT 24.21 (min), and compound 25; Myricetin 3‐*O*‐galactoside [*m/z* 479.0735] was detected at RT 21.50 (min), both in the negative mode of ionization [M‐H]^−^. Further MS/MS analysis confirmed the compounds (23 and 25) through product ions [*m/z* 447], [*m/z* 317], and [*m/z* 285], resulting in loss of sequential single (162 Da) and double (324 Da) hexosyl moieties, respectively, indicating the breakdown of kaempferol. Compound 24 [Quercetin 3′‐*O*‐glucuronide] with observed mass [*m/z* 477.0682] and compound 25 [Quercetin 3‐*O*‐xylosylrutinoside], [*m/z* 741.1418] were quercetin compounds, a prominent class of flavonoids found in plants and fruits. Compounds were further identified through MS^2^ mass spectrum; compound 24 resulted in product ions [*m/z* 301] through the removal of glucuronide (176 Da), and compound 26 resulted in product ions [*m/z* 479] and [*m/z* 317], resulting from the loss of two pentose moieties and one hexose moiety from precursor ions (Nebieridze et al. 2017; Elshamy et al. 2024). Results are supported by a previous study that identified potential bioactive polyphenols from wild edible flowers, including *Tagetes*, and found related phenolics, carotenoids, and flavonoids in pigment‐rich extracts with potential antioxidant activities (Rivas‐García et al. 2023). Variations in compounds might result from growing conditions, differences in varieties, and extraction methods (Hegde et al. 2023; Liu et al. 2024).

Other polyphenols; tyrosol [*m/z* 139.0743], esculin [*m/z* 339.0245] and coumarin [*m/z* 147.0431] were detected in different extract samples including MFHU, MFAC, MFHC, MFAU, and MFEU. Coumarin was identified in positive mode of ionization [M‐H]^+^ with observed [*m/z* 147.0431] at [RT 3.31 (min)] exhibited product ions [m/z 103, 91] in the mass spectrum [MS^2^] resulting through the removal of CO_2_ (44 Da) and 2CO (56 Da) (Liu et al. 2024). In our analysis, no metabolites corresponding to condensed tannins were detected that matched the reference library spectra while in hexane samples Total condensed tannins were observed (Table 2). This discrepancy may also be attributed to the sample preparation process for LC–MS, where filtration and dilution steps could potentially mask or reduce the ionization efficiency of certain high‐molecular‐weight compounds such as condensed tannins. In contrast, spectrophotometric assays (e.g., TCT) are more sensitive to the presence of these polymeric compounds, which could explain the observed difference. Further, HPLC can be performed in future studies to confirm presence and estimation of total condensed and to provide deep understanding of other polyphenolic compounds in medicinal plants (Yu et al. 2021).

### 3.6 HPLC‐DAD Quantification of Phenolic Compounds of *Tagetes* Flower Extracts

Quantification of bioactive compounds including phenolics, flavonoids, and carotenoids of freeze‐dried extract samples was performed through HPLC‐DAD. Presented results (Table 6) showed the distribution of various bioactive compounds at various wavelengths (λ) and retention times (RT), including phenolic acids [gallic acid (RT 5.76 min; 280 λ), coumaric acid (RT 38.36 min; 313 λ), syringic acid (RT 29.33 min; 280 λ)], flavonoids [quercetin (RT 54.63 min; 350 λ), catechin (RT 22.34 min; 280 λ), and kaempferol (RT 56.77 min; 350 λ)], and carotenoids [lutein (RT 16.03 min; 450 λ), zeaxanthin (RT 7.24 min; 450 λ), and fucoxanthin (RT 4.79 min; 450 λ)] in different solvent extract samples.

**TABLE 6:** HPLC‐DAD Quantification of Phenolic Compounds of *Tagetes* Flower Extracts.

Samples		Phenolic acids			Flavonoids			Carotenoids			Sum of compounds
		Gallic acid (mg/g)	Coumaric acid (mg/g)	Syringic acid (mg/g)	Quercetin (mg/g)	Catechin (mg/g)	Kaempferol (mg/g)	Lutein (mg/g)	Zeaxanthin (mg/g)	Fucoxanthin (mg/g)	
Conventional Solvent Extraction (CSE)	MFAC	43.60 ± 0.55^d^	2.48 ± 0.52^bc^	—	7.46 ± 0.21^b^	2.57 ± 0.15^a^	1.77 ± 0.07^b^	1.45 ± 0.05^ab^	—	—	57.55
	MFEC	52.89 ± 0.30^b^	3.36 ± 0.30 ^ab^	—	10.45 ± 0.07^a^	2.34 ± 0.33^a^	1.92 ± 0.04^ab^	1.33 ± 0.02^b^	—	—	70.36
	MFHC	19.51 ± 0.37^f^	1.67 ± 0.04^c^	—	2.50 ± 0.04^c^	—	—	0.74 ± 0.01^c^	0.65 ± 0.01^c^	—	25.06
Ultrasound Assisted Extraction (UAE)	MFAU	47.57 ± 0.51^c^	3.26 ± 0.15 ^ab^	1.84 ± 0.20^a^	10.70 ± 0.66^a^	2.81 ± 0.15^a^	1.87 ± 0.04^ab^	1.71 ± 0.10^a^	2.18 ± 0.16^a^	0.65 ± 0.01^b^	70.71
	MFEU	60.61 ± 0.69^a^	4.28 ± 0.08^a^	1.62 ± 0.08^a^	10.64 ± 0.21^a^	2.92 ± 0.09^a^	1.96 ± 0.01^a^	1.81 ± 0.23^a^	2.24 ± 0.05^a^	1.18 ± 0.08^a^	85.28
	MFHU	29.42 ± 0.48^e^	1.71 ± 0.08^c^	0.83 ± 0.23^b^	3.13 ± 0.03^c^	—	—	1.20 ± 0.08^b^	1.26 ± 0.01^b^	—	37.54

*Note:* The data shown in the table as mean ± standard deviation (*n* = 3); lettering (a, b, c, d, e, f) indicated the significant difference in the means (*p* < 0.05) using a one‐way analysis of variance (ANOVA) and Tukey's HSD test. Sample abbreviations are explained at Table 1.

The highest total concentration of bioactive compounds was observed in ultrasound extract samples MFEU followed by MFAU extract. Among phenolic acids, gallic acid and coumaric acid were found in all UAE extract samples while syringic acid was not detected in CSE samples. Other important classes of polyphenolics detected were flavonoids including Quercetin, catechin, and kaempferol (Table 6). The highest concentration of Quercetin was found in the ethanolic extract of ultrasound‐treated sample MFAU and MFEU (10 mg/g) while the lowest concentration was observed in conventional solvent extract MFHC (2.50 mg/g). Among carotenoids lutein, zeaxanthin higher concentrations were detected in UAE samples than in CSE. The highest lutein concentration was detected in MFEU (1.81 mg/g) and the lowest was observed in MFHC (0.74 mg/g). This may be due to the loss of bioactive compounds that are thermolabile and can be easily degraded at higher temperatures (Jalali‐Jivan et al. 2021). Lutein and zeaxanthin concentrations detected at specific wavelengths (430–575 nm) and retention time (RT) are also in line with previous studies on quantification of various compounds of *Tagetes* (Kashyap et al. 2022; Liu et al. 2011).

Current research findings, also supported by previous literature, reported that efficient extraction of carotenoids is highly dependent on appropriate solvent selection (Becerra et al. 2020). Results showed that marigold hexane extracts resulted in the least content of lutein, and other carotenoids were not detected in some of the CSE extracts and hexane extracts of UAE. This may also be due to the higher polarity of xanthophylls than the extracting solvent (hexane), making it more suitable for non‐polar carotenoids (like carotenes) than polar xanthophylls. The presented results are also supported by a previous study that reported that acetone resulted in the highest lutein recovery due to its strong ability to dissolve polar carotenoids (Low et al. 2020). Evidence also reported that the polar functional groups of lutein and zeaxanthin make them more soluble and extractable in polar solvents like acetone, ethanol, and tetrahydrofuran than in hexane (Saini et al. 2023; Saini and Keum 2018). Morón‐Ortiz et al. reported that organic solvents pose a high risk (health and environmental) and result in relatively low carotenoid yield upon extraction (Morón‐Ortiz et al. 2024).

The presence of phytochemicals like phenolic acids in marigold extract samples also reflects that the extract has good pharmaceutical and nutritional properties. Gallic acid (C_7_H_6_O_5_) is a natural phenolic compound mostly present in medicinal plants and is known for its pharmacological activities like anti‐inflammatory, cardiovascular, gastrointestinal, neuropsychological, and metabolic functions (Kahkeshani et al. 2019). Quercetin (C_15_H_10_O_7_) is an important member of flavonoids, which is highly recognized for its antioxidant, anti‐cancerous, and antiaging properties (Shi et al. 2023). Lutein (C_40_H_56_O_2_) consists of a hydroxyl group and has the ability to cross the blood ocular barrier (BOB) and helps to control age‐related macular disorders (Duan et al. 2024).

## 4 Conclusion

This study elucidated that ultrasonication extracted higher phenolic content and antioxidant capacity in *Tagetes* flower extracts when compared to conventional methodology with 70% ethanol and acetone. The combined process of freeze drying and ultrasonication significantly improved the phenolic content and antioxidant capacity of *Tagetes* flowers, as evaluated using in vitro experiments. Component analysis showed a significant positive correlation among various variables of phenolic and antioxidant assays. Additionally, phenolic compounds identification and characterization by LC MS/MS analysis found various polyphenolic compounds that have previously demonstrated potential health benefits. Further, HPLC quantification confirmed higher phenolic content (sum of compounds) in freeze‐dried UAE extracts, including important polyphenolic compounds such as gallic acid, coumaric acid, quercetin, lutein, and zeaxanthin. These findings on phenolic compounds will help us further investigate in vitro bioaccessibility through the stimulated digestive model for the utilization of these bioactive compounds for functional or nutraceutical food formulation.

## Author Contributions

**Ayesha Siddiqa:** conceptualization (equal), formal analysis (equal), methodology (equal), writing – original draft (equal), writing – review and editing (equal). **Adnan Khaliq:** conceptualization (equal), methodology (equal), project administration (equal), writing – review and editing (equal). **Muhammad Tauseef Sultan:** conceptualization (equal), data curation (equal), methodology (equal), software (equal), supervision (equal), writing – review and editing (equal). **Muhammad Farhan J. Chugthai:** project administration (equal), supervision (equal), writing – review and editing (equal). **Samreen Ahsan:** formal analysis (equal), project administration (equal), software (equal), supervision (equal), writing – review and editing (equal). **Waseem Khalid:** data curation (equal), methodology (equal), software (equal), visualization (equal), writing – review and editing (equal). **Hafiz Suleria:** formal analysis (equal), funding acquisition (equal), methodology (equal), supervision (equal), writing – original draft (equal), writing – review and editing (equal).

## Conflicts of Interest

The authors declare no conflicts of interest.

## Data Availability

The data that support the findings of this study are available from the corresponding author upon reasonable request.
